# Effect of troxerutin on apelin-13, apelin receptors (APJ), and ovarian histological changes in the offspring of high-fat diet fed rats

**DOI:** 10.22038/ijbms.2019.34158.8123

**Published:** 2019-06

**Authors:** Keyvan Mehri, Seyed Mahdi Banan Khojasteh, Banan Khojasteh Seyed Mahdi, Fereshteh Fereshteh, Zohreh Zavvari Oskuye, Hadi Ebrahimi, Roghaye Diba, Parvin Bayandor, Maryam Hosseindoost, Shirin Babri

**Affiliations:** 1 Drug Applied Research Center, Tabriz University of Medical Sciences, Tabriz, Iran; 2 Department of Animal Biology, Faculty of Natural Sciences, University of Tabriz, Iran; 3 Neurosciences Research Center (NSRC), Tabriz University of Medical Sciences, Tabriz, Iran

**Keywords:** Apelin-13, APJ receptor, Maternal high-fat diet, Ovarian development, Troxerutin

## Abstract

**Objective(s):**

Maternal high-fat diet (HFD) consumption has been linked to metabolic disorders and reproductive dysfunctions in offspring. Troxerutin (TRO) has anti-hyperlipidemic, anti-oxidant, and anti-inflammatory effects. This study examined the effects of TRO on apelin-13, its receptors mRNA and ovarian histological changes in the offspring of HFD fed rats.

**Materials and Methods:**

Female Wistar rats were randomly divided into control diet (CD) or HFD groups and received these diets for eight weeks. After mating, dams were assigned into four subgroups: CD, CD + TRO, HFD, and HFD + TRO, and received their respective diets until the end of lactation. Troxerutin (150 mg/kg/day) was gavaged in the CD + TRO and HFD + TRO groups during pregnancy. On the postnatal day (PND) 21 all female offspring were separated and fed CD until PND 90. On PND 90 animals were sacrificed and ovarian tissue samples were collected for further evaluation.

**Results:**

Results showed that HFD significantly decreased serum apelin-13 in the female offspring of the HFD dams, which was significantly reversed by TRO. Moreover, real-time polymerase chain reaction (PCR) analysis revealed that TRO treatment significantly decreased the ovarian mRNA expression of the apelin-13 receptor in the troxerutin-received offspring. Furthermore, histological examination revealed that TRO increased the number of atretic follicles in the ovaries of HFD+TRO offspring.

**Conclusion:**

Maternal high fat feeding compromises ovarian health including follicular growth and development in the adult offspring and troxerutin treatment improved negative effects of maternal HFD on the apelin-13 level and ovarian development of offspring.

## Introduction

Obesity is a growing public health problem worldwide that affects all social classes of all ages (1). A growing body of evidence shows that maternal nutrition has a long-term adverse impact on the early development and health of offspring (2, 3). Maternal obesity during gestation is also linked to offspring obesity which often extends across the lifespan (4). Several epidemiologic and experimental studies have demonstrated that obesity is associated with reproductive dysfunctions including infertility, ovulatory dysfunction, and hypogonadism (5). Animal studies also showed that exposure to HFD during pregnancy has deleterious effects on follicular development and growth of the ovaries (3), and alters estrous cycle in offspring (6). 

Adipokines referr to secreted factors from the adipose tissue that are implicated in insulin resistance, inflammation, energy metabolism, and normal function of the reproductive system and fertility (7). Apelin, a biologically active peptide belongs to the adipokines family, is considered an endogenous ligand for G-protein coupled receptor APJ (8-11). Several active fragments of apelin including apelin-13, apelin-17, and apelin-36 have been extracted; apelin-13 is the most potent activator of the cells expressing APJ and has higher affinity to APJ receptors (12, 13).

Apelin and its receptor APJ are widely expressed in the central nervous system (CNS), as well as in the various peripheral tissues including lung, heart, kidney, white adipose tissue, testicles, and uterus (14). Apelin/APJ receptors have an emerging role in the physiological regulation of the cardiovascular system, metabolism, cell proliferation and apoptosis, and immune system (15, 16). Several recent studies demonstrated that plasma apelin concentration is increased in the obese mice during pregnancy, and decreased in women with polycystic ovary syndrome (8, 17). In addition, previous reports confirmed the presence of apelin and APJ receptors in the reproductive organs for example testis and ovaries, as well as in the brain sections where gonadotropin-*releasing* hormone (GnRH) is released indicating that apelin is a modulatory factor in the reproductive system (12, 14, 18). However, there is no information about the effects of these alterations in the offspring. Therefore, it is necessary to investigate the influence of maternal HFD on the reproduction health of offspring.

Troxerutin, commonly known as vitamin P4, is a derivate of natural bioflavonoid and one of the constituents of tea, coffee, and different kinds of fruits and vegetables (19). Troxerutin possesses several pharmacological properties such as strong antioxidant, anti-inflammatory, anti-hyperlipidemic, hepatoprotective, and neuroprotective (20-24). In addition, troxerutin has a protective effect against testicular toxicity induced by Nickel in rats (25). Recent reports from our laboratory have shown that troxerutin attenuated HFD-induced spatial memory impairments in the male offspring of HFD fed dams (26).

Based on the above, the objective of the present study was to examine the effects of troxerutin administration during pregnancy on serum apelin-13 level and mRNA expression of its receptors and ovarian histological changes in the offspring of HFD fed rats.

## Materials and Methods

***Experimental design***

Three-weeks-old female Wistar rats (n=40) were obtained from the Animal House of Tabriz University of Medical Sciences and housed three per cage at room temperature (22–25 ^°^C) with 12:12 hr light/dark cycles. Animals had access to food and water *ad libitum*. All protocols and guidelines issued by the Ethical Committee of Tabriz University of Medical Sciences regarding the protection and dissection of animals in research were firmly followed.

Following one week for adaptation, animals were randomly divided into two groups of 20 rats and received control diet (CD) (14.7% lipids, 33.0% protein, 52.2% carbohydrate)or high-fat diet (HFD) (52.0% lipids, 27.1% protein, 20.9% carbohydrate) for 8 weeks. For mating, animals were housed with adult males rats fed control diet overnight. Following confirmation of pregnancy by investigating vaginal smears for the presence of sperm, *pregnant *rats were kept in separate cages and randomized to four subgroups:* CD, *CD+TRO, *HFD,* and HFD+TRO. All groups continued to receive their respective diet until the end of lactation. Troxerutin (Merck, Germany) 150 mg/kg/day was gavaged to the troxerutin-received groups during pregnancy. Female offspring of all groups were weaned on PND 21 and kept separately in their respective maternal groups and fed CD until PND 90.

**Figure 1:**
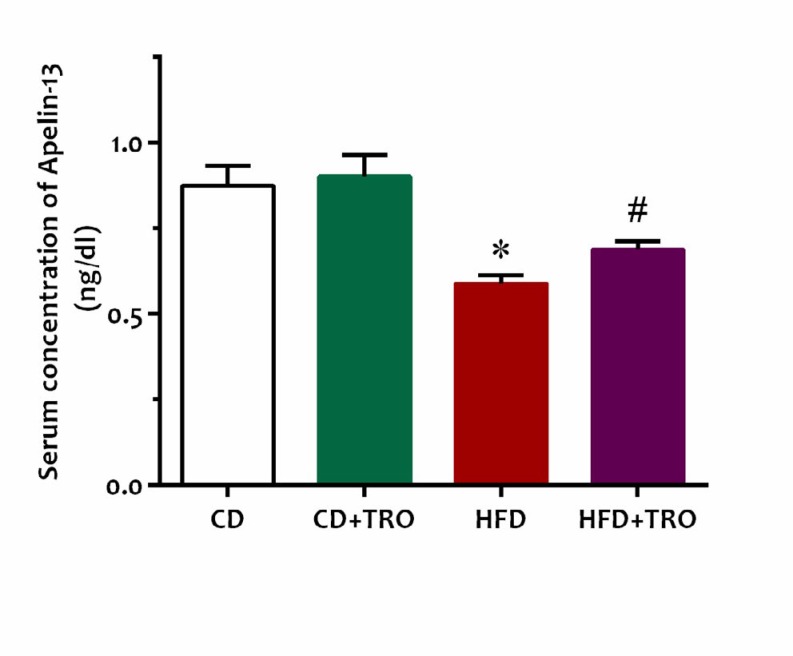
Effect of maternal high fat diet exposure and troxerutin treatment during pregnancy on offspring serum apelin-13 concentration. Data are expressed as Mean±SEM (n=8). One-way ANOVA followed by Tukey’s *post hoc* test; *P<0.05 vs CD group, #*P*<0.05 vs HFD group. [CD: control diet, CD+TRO: control diet + Troxerutin, HFD: High-fat diet; HFD+TRO: High-fat diet + Troxerutin]

**Figure 2:**
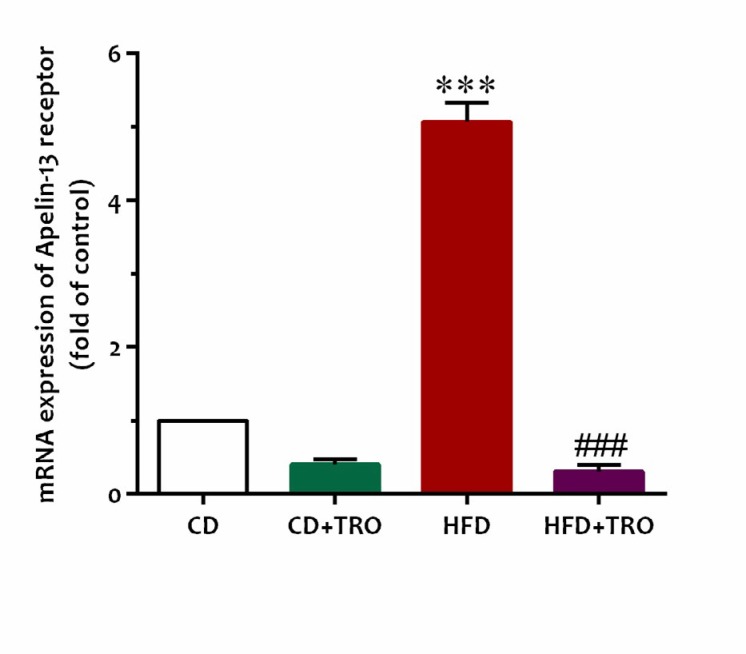
Relative expression of apelin receptor (APJ) in the ovaries of adult offspring of HFD/CD-fed dams. Data are expressed as Mean± SEM (n=6). Kruskal-Wallis followed by Mann-Whitney *post hoc *test; ***P<0.001 vs CD group, ###*P*<0.001 vs HFD group. [CD: control diet, CD+TRO: control diet+Troxerutin, HFD: High-fat diet, HFD+TRO: High-fat diet+Troxerutin]

**Figure 3:**
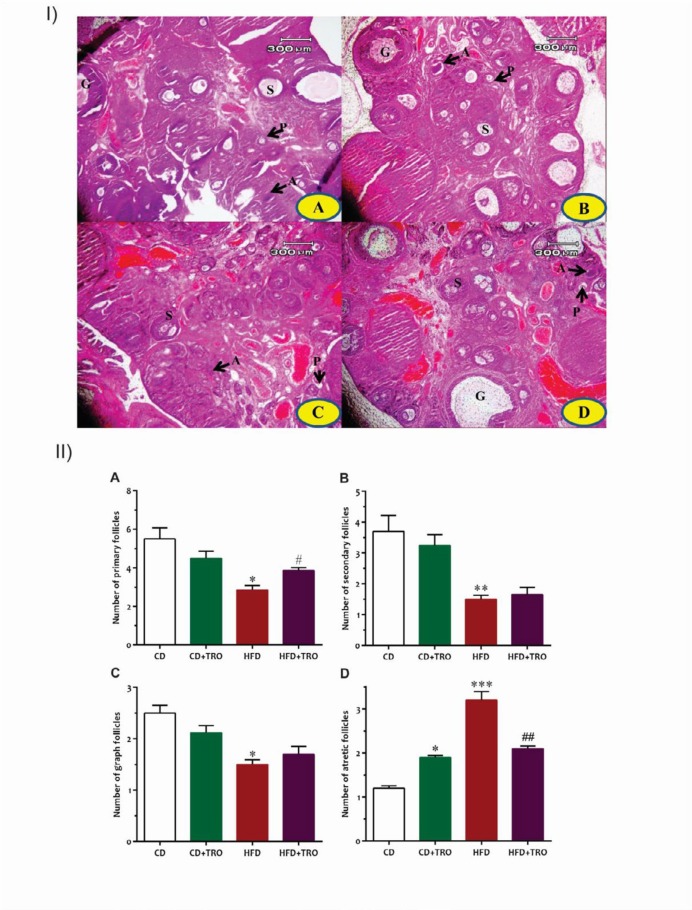
(I) Light micrographs of cross-sections of the ovaries of offspring stained with H&E (40x) [P: Primary follicle; S: Secondary follicle; G: Graafian follicle; A: Atretic follicle]. (II) Effect of maternal high-fat diet exposure and treatment with troxerutin during pregnancy on the number of primary follicles (A), secondary follicles (B), Graafian follicles (C), and atretic follicles (D) in the ovary. Data are expressed as Mean±SEM (n=8). One-way ANOVA followed by Tukey’s post hoc test; *P<0.05, **P<0.01 vs CD group, #*P*<0.05 vs HFD group. [CD: control diet, CD+TRO: control diet + Troxerutin, HFD: High-fat diet, HFD+TRO: High-fat diet +Troxerutin]

***Sampling***

At the end of the procedures, all female offspring were anesthetized with intraperitoneal injection of ketamine (80 mg/kg) and xylazine (12 mg/kg). Before removing the ovaries, vaginal examination was performed to determine the period of the cycle. After confirming the diestrus period of the cycle (27, 28), the ovaries were excised and weighed by a digital scale. Right ovary was washed with saline and kept in 10% buffer formalin for histological examination, while the left one was immediately frozen with liquid nitrogen and then kept at -70 ^°^C for molecular examination. Blood samples were collected from the heart and centrifuged at 4000 rpm for 15 min and serum samples were separated and kept at -70 ^°^C for apelin-3 measurement.

***Histological analysis***

For histological study, ovaries were fixed in formalin 10% solution, dehydrated in ethyl alcohol and then cleared in xylol. Paraffin-embedded ovarian tissues were cut at 7 µm thickness using a microtome and stained with hematoxylin-eosin (H&E). Seven characteristic sections with 200 nm interval between sections were selected from each ovary. The number of primary, secondary, Graafian and atretic follicles were counted in the prepared slides from each block by a blinded experimenter to the treatment groups using a light microscope (Nikon, Tokyo, Japan) at 400X total magnification.

***Assessment of apelin serum concentration***

Serum concentration of apelin-13 was measured using commercially available rat-specific apelin-13 enzyme-linked immunosorbent assay (ELISA) kit (EAST BIOPHARMA) according to the manufacturer’s instructions.

***Total RNA extraction and real-time PCR***

Total RNA was isolated from the ovarian tissue samples using the RNX-Plus solution kit (Cinagen Co. Iran) according to the manufacturer’s instructions. Quantity and purity of the RNA were measured using a NanoDrop 1000 Spectrophotometer (Thermo Scientific, USA). Reverse transcription of the RNA samples into cDNA was performed using the PrimerScript RT Master Mix Perfect Real-Time Kit (Takara Bio Inc.). Real-time PCR was carried out using SYBR Green PCR Master Mix (Takara Bio, Shiga, Japan) in a total volume of 25 µl on a real-time PCR instrument (RotorGene 3000). PCR primer sequences for APJ receptor and β-actin (housekeeping) were as follows: APJ (Forward 5^΄^- CCTGGCTTGATGCAGTTGGA-3^΄^, Reverse 5^΄^- TCTGGCCTGAGACATGCAGAG-3^΄^; β-actin (Forward 5^΄^-TACAGCTTCACCACCACAGC-3^΄^, Reverse 5^΄^-ATGCCACAGGATTCCATACC-3^΄^). The relative quantity of mRNA for each gene was calculated in relation to its threshold cycle (Ct) compared to the Ct of the internal control gene (β-actin). Relative expression of the target gene was calculated using the 2^-ΔΔCt^ method as follows: 

2^-[(Ct APJ gene – Ct β-actin) experimental - (Ct APJ gene -Ct β-actin) Control]^ (29).

***Data analysis***

Data were expressed as mean±standard error of the mean (SEM). Statistical analysis was performed by SPSS 16 using analysis of variance (ANOVA) followed by Tukey’s *post hoc* test. Kruskal-Wallis followed by Mann-Whitney *post hoc* test was used for analysis of real-time PCR results. *P*-value <0.05 level was considered statistically significant.

## Results

***Effect of maternal HFD and TRO treatment on serum apelin-13 level in the offspring***

Serum apelin-13 concentration in the offspring of HFD dams significantly decreased in comparison with the offspring of the CD and CD+TRO fed dams (*P*<0.05, Figure 1). Conversely, troxerutin treatments during pregnancy significantly (*P*<0.05) increased serum apelin-13 levels in the HFD+TRO offspring group as compared to the HFD group.

***Effect of maternal HFD and TRO treatment on ovarian mRNA expression of APJ receptor in the offspring***

The results of real-time PCR showed that ovarian mRNA expression of APJ, apelin-13 receptor, was markedly up-regulated in the HFD offspring in comparison with the offspring of the CD group (*P*<0.001, Figure 2). Nevertheless, chronic administration of troxerutin during gestation significantly (*P*<0.001) down-regulated mRNA expression of the APJ receptor in the offspring of the HFD+ TRO group in comparison with the HFD group.

***Effect of maternal HFD and TRO treatment on ovarian morphology in offspring***

Histological examination showed that maternal HFD affects the number of primary (Figure 3A), secondary (Figure 3B), graafian (Figure 3C), and atretic follicles (Figure 3D) in the ovaries of adult offspring. The results of one-way ANOVA also revealed that maternal HFD significantly decreased the number of primary (*P*<0.05), secondary (*P*<0.01), and de Graaf’s follicles (*P*<0.05), and increased (*P*<0.001) the number of atretic follicles in the ovaries of adult offspring as compared to the offspring of CD and CD+TRO groups. Conversely, troxerutin significantly increased (*P*<0.05) the number of primary follicles in the ovaries of the HFD+TRO offspring, and decreased (*P*<0.01) the number of atretic follicles in the ovaries of adult offspring in comparison to HFD offspring.

## Discussion

Results of the current study demonstrated that maternal HFD (pre-pregnancy, pregnancy, and lactation periods) caused significant changes in the serum levels of apelin-13 and its receptor mRNA expression, APJ, in the ovary tissue of offspring. Moreover, offspring of HFD fed dams represented a remarkable reduction in the number of primary, secondary, and Graafian follicles, as well as an increase in the number of atretic follicles compared to the CD offspring indicating that HFD can affect the reproductive potential of the adult female offspring. Nevertheless, troxerutin treatment during pregnancy increased serum apelin-13 and down-regulated apelin-13 receptor mRNA expression in the ovarian tissue of the offspring of HFD fed mothers. Additionally, troxerutin increased the number of primary, secondary, and graph follicles, and decreased atretic follicles.

Previous studies showed that HFD feeding for 15 days is associated with elevated adipose tissue, decreased adiponectin secretion from the adipose tissue, and reproductive disorders both in the male and female rats (30, 31). A recent study also demonstrated that adipocytokine promoter epigenetic can be affected by maternal HFD exposure resulting in unusual adipocytokine levels in the offspring (32). Moreover*, in vivo* studies showed that apelin expression is markedly regulated by nutritional status, suppressed by fasting and restored by refeeding, as well as insulin (11, 33). Exposure to high fatty acid levels leads to insulin resistance and hyperinsulinemia, which subsequently rises apelin release from the adipose tissue (33, 34). Moreover, there is a direct relationship between apelin secretion and body mass index (BMI), and obese patients have higher apelin plasma concentration than normal-weight controls (35, 36). In the present study, we also found low levels of apelin-13 in the serum of HFD fed dam offspring as compared to the CD group. Apelin has an anti-inflammatory property which restricts neuroinflammatory processes (37-39). According to a previous study, apelin can suppress pro-inflammatory cytokine expression such as tumor necrosis factor-alpha and interleukin (IL)-1β protein  (40). On the other hand, previous studies revealed an association between apelin levels and inflammatory, as well as oxidative stress markers, and apelin expression is enhanced by inflammatory factors such as TNF-α (10, 41, 42). On the other hand, troxerutin treatment increased serum levels of apelin-13 in the offspring of HFD-received troxerutin dams. Likewise, Zhang *et al.* (43) reported that troxerutin inhibits inflammatory cytokine release and decreases adiponectin levels in HFD-received mice. Furthermore, troxerutin can prevent obesity by improving the insulin signaling pathway and returns blood glucose, fatty acids, and cholesterol levels to normal levels (24). 

Findings of the present study also demonstrated that maternal HFD causes a significant up-regulation in apelin receptor mRNA expression in the ovary of their offspring. This enhanced receptor gene expression may result in reduced apelin-13 serum level via feedback inhibition (44). Clarke *et al.* (45) also reported that HFD up-regulated the expression of the APJ receptor, and central administration of apelin down-regulated these receptors in the hypothalamus. The findings of the current study also demonstrated that troxerutin (150 mg/kg) for 21 days in the HFD pregnant dams remarkably reduced expression of apelin-13 receptor mRNA in the ovarian tissue of offspring. 

Reproductive function is sensitive to various environmental conditions such as seasonal change, exposure to different toxins, and nutritional status (46, 47). Since the primordial germ cells of the fetal ovary are susceptible to gestational environmental insults, maternal HFD exposure may have a negative impact on reproductive consequences in the offspring (48). Evidence shows that both maternal high fat feeding and postnatal HFD can modify follicular development and increase follicular atresia in offspring born to HFD fed dams (49, 50). Long-term (two months) cafeteria diet has been shown to damage the ovulatory process and result in follicular cysts in rats (51). Furthermore, HFD for 20 weeks impairs cell cycle and prompts apoptosis in granulosa cells during follicular development (52). Another study (53) also found that maternal HFD during pregnancy and lactation leads to follicular impairments and increased follicular atresia. Histological analysis of the current study also revealed that HFD led to abnormal ovarian morphology and decreased the number of primordial, antral, as well as Graafian follicles, while increasing it in the ovaries of offspring. Furthermore, the number of atretic follicles considerably increased in the offspring of HFD fed dams. It is likely that HFD increased atretic follicles via up-regulation of ovarian cell cycle inhibitors, augmentation of granulosa cells (GCs) apoptosis (52), or modifications of the expression of genes involved in growth, development, and apoptosis of follicles in the ovary (49). Xu *et al*. (54) reported that exposure to maternal HFD deteriorates ovarian health in offspring of pigs via ovarian oxidative stress and cell apoptosis.

Results obtained from this study showed that administration of troxerutin in the HFD + TRO group during pregnancy decreased the number of atretic follicles in the offspring in comparison with offspring of the HFD group. Moreover, these results were associated with increased serum apelin-13 levels. Numerous studies have reported that apelin and its receptors are widely expressed in the reproductive organs in humans and rat, indicating apelin may participate in the regulation of the reproduction system (14, 55, 56). Moreover, previous evidence supports the anti-inflammatory, anti-oxidative, and anti-apoptotic effects of troxerutin (20, 21, 57, 58). Our group recently demonstrated that troxerutin treatment increased hippocampal and serum levels of apelin in the male HFD offspring (59). Therefore, it is likely that troxerutin treatment inhibits the deleterious effect of maternal HFD on offspring ovarian health through increasing circulating apelin-13 levels and inhibiting oxidative stress and apoptosis cell death.

## Conclusion

Overall, the results of the current study showed decreased serum apelin and an increase in apelin/APJ mRNA expression of ovarian tissue in offspring of HFD fed dams, and these changes were restored by troxerutin treatment. Maternal HFD exposure also had deleterious consequences on follicular growth and development in the adult offspring ovaries, which partially improved by troxerutin treatment. More studies are required to identify the exact mechanism of the troxerutin effect in this context. 
